# *Akkermansia muciniphila* Alleviates *Enterococcus faecalis*-Exacerbated Alcoholic Liver Injury by Modulating Gut Microbiota and Barrier Function

**DOI:** 10.3390/ijms27125474

**Published:** 2026-06-17

**Authors:** Xin Sui, Songhui Feng, Weitao Wang, Xin Zhang, Yang Liu, Nan Peng

**Affiliations:** 1 School of Biological Science and Technology, University of Jinan, Jinan 250022, China; bio_suix@ujn.edu.cn; 2 National Key Laboratory of Agricultural Microbiology, Hubei Hongshan Laboratory, College of Life Science and Technology, Huazhong Agricultural University, Wuhan 430070, China; fengsh@webmail.hzau.edu.cn (S.F.); mq3189@webmail.hzau.edu.cn (W.W.); xinzhang2006@outlook.com (X.Z.); liuyangzgsd@163.com (Y.L.)

**Keywords:** *Akkermansia muciniphila*, alcoholic liver disease, *Enterococcus faecalis*

## Abstract

Cytolysin-positive *Enterococcus faecalis* is a key pathogen in severe alcoholic hepatitis, yet the mechanisms through which it worsens disease and possible therapeutic strategies remain poorly understood. This study aimed to clarify the pathogenic effects of *E. faecalis* in acute alcohol-associated liver disease (ALD) and to assess the protective potential of *Akkermansia muciniphila* (Akk11) against this pathogen. Using a mouse model of acute ethanol gavage, animals received *E. faecalis* and/or Akk11 under prophylactic or therapeutic regimens. Assessments included liver injury markers, histopathology, lipid profiles, inflammatory cytokines, gut barrier integrity, and gut microbiota composition. *E. faecalis* exacerbated ethanol-induced hepatic steatosis and injury, showing a paradoxical effect: it increased histological damage while lowering circulating LPS and transaminases. This was linked to upregulated hepatic autophagy (increased Atg7) and reduced cholesterol, yet it promoted neutral lipid accumulation. Importantly, *E. faecalis* aggravated gut dysbiosis by markedly enriching the pro-inflammatory pathobiont *Helicobacter typhlonius* and impairing colonic barrier function. Intervention with Akk11 alleviated liver injury, reduced lipid accumulation and oxidative stress, and restored cytokine balance. Akk11 also strengthened gut barrier integrity, lowered serum endotoxin, and beneficially reshaped the microbiota. Prophylactic administration was particularly effective, normalizing the *Firmicutes*/*Bacteroidota* ratio, suppressing *H. typhlonius*, and enriching beneficial *Bacteroides sartorii*. This study confirms the pathogenic role of *E. faecalis* in acute ALD and establishes *A. muciniphila* (Akk11) as a promising microbiota-targeted therapy, which protects against liver injury by reinforcing the gut barrier, selectively modulating microbiota, and reducing inflammation, with prophylactic administration showing superior efficacy.

## 1. Introduction

Alcohol-associated liver disease (ALD) is a leading cause of liver-related mortality globally, representing a significant public health burden with limited therapeutic options [1,2]. Characterized by a multifactorial pathogenesis, ALD involves not only the direct hepatotoxic effects of ethanol and its metabolites but also oxidative stress, cell death, and gut microbiome dysbiosis [3,4,5,6]. The gut–liver axis has been increasingly identified as playing a central role in disease development and progression [7,8,9]. Ethanol consumption disrupts intestinal homeostasis, leading to a state of microbial dysbiosis characterized by a reduction in beneficial taxa and an overgrowth of potentially harmful microorganisms [10,11]. This dysbiosis, in turn, impairs the function of the intestinal epithelial barrier [12,13]. The compromised barrier facilitates the translocation of microbial products, such as lipopolysaccharide (LPS), into the portal circulation. Upon reaching the liver, these microbial mediators activate hepatic immune cells, primarily Kupffer cells, triggering a cascade of pro-inflammatory responses that exacerbate hepatic steatosis, inflammation, and injury [14]. Recent comprehensive reviews have summarized these intricate mechanisms, consistently highlighting the considerable potential of microbiota-targeted interventions as a novel therapeutic strategy for ALD [15,16,17,18,19,20].

A pivotal development in the field has been the identification of specific pathobionts that drive disease severity. Notably, *Enterococcus faecalis*, particularly strains capable of producing the pore-forming toxin cytolysin, has been identified as a key pathogen in patients with severe alcoholic hepatitis. Its presence in these patients is strongly correlated with increased mortality and worse clinical outcomes [21]. Evidence from humanized mouse models has demonstrated a causal relationship: transplantation of cytolysin-positive *E. faecalis* from patients into mice exacerbated ethanol-induced liver injury, while subsequent targeted elimination of this bacterium using specific bacteriophages significantly attenuated the hepatic damage [21]. These findings position *E. faecalis* as a promising therapeutic target. However, the precise mechanisms by which *E. faecalis* influences host physiology under conditions of acute ethanol exposure—such as its specific impact on intestinal permeability, its interaction with the mucus layer, and its synergistic or antagonistic relationships with broader shifts in the microbiota community (e.g., the commonly observed increase in the *Firmicutes*/*Bacteroidota* ratio)—remain areas of active investigation.

Concurrently, the exploration of next-generation probiotics (NGPs) offers a complementary therapeutic approach. Among these, *Akkermansia muciniphila* has garnered significant attention for its ability to enhance intestinal epithelial integrity, modulate systemic immune responses, and ameliorate metabolic endotoxemia [22,23,24,25]. *A. muciniphila*, a mucin-degrading bacterium residing in the intestinal mucus layer, contributes to gut barrier function through mechanisms such as promoting mucus layer renewal and stimulating the production of antimicrobial peptides. Although a substantial body of evidence supporting its benefits originates from studies on metabolic diseases like obesity and type 2 diabetes, the barrier-strengthening and anti-inflammatory properties of *A. muciniphila* suggest considerable potential in attenuating alcohol-induced disruption of the gut–liver axis [26,27]. Nevertheless, its efficacy in the specific context of acute ethanol challenge, particularly in an environment dominated by a pathobiont like *E. faecalis*, has not been thoroughly investigated, leaving a critical gap in understanding the interplay between specific probiotics and pathogens in ALD.

This study employed a mouse model of acute ethanol exposure to investigate the mechanisms by which *E. faecalis* exacerbates hepatic injury and alters the gut microbiota, and to evaluate the efficacy of *A. muciniphila* (strain Akk11) administered either prophylactically or therapeutically against these effects. Using an integrated approach—including analysis of organ indices, assessment of lipid peroxidation, profiling of inflammatory cytokines, quantification of serum LPS, histopathological evaluation, and 16S rRNA gene sequencing—a comprehensive analysis of microbe–host interactions in ALD was performed. The results clarify the pathogenic role of *E. faecalis* and support the therapeutic potential of *A. muciniphila* as a microbiota-based intervention for ALD.

## 2. Results

### 2.1. Enterococcus faecalis Exacerbates Acute Ethanol-Induced Liver Injury

**Figure 1:**
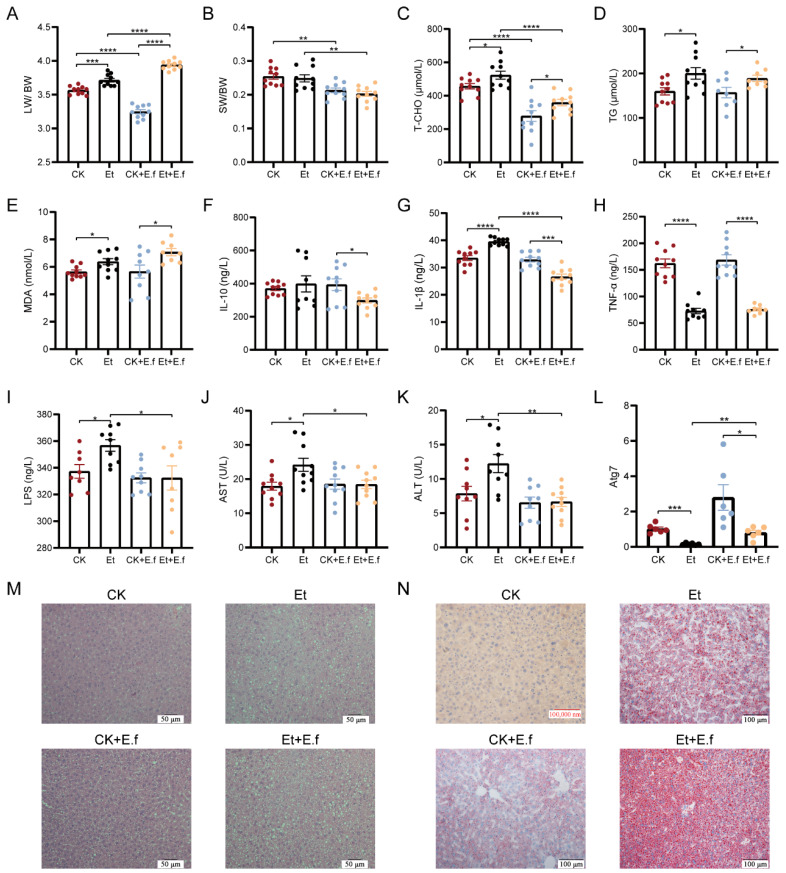
*Enterococcus faecalis* exacerbates alcoholic liver injury in mice. (**A**) Liver index (liver weight/body weight ratio); (**B**) Spleen index; hepatic (**C**) total cholesterol; (**D**) triglycerides (TG); (**E**) malondialdehyde (MDA); (**F**) IL-10; (**G**) IL-1β; (**H**) TNF-α levels in liver tissue; serum (**I**) LPS; (**J**) AST; (**K**) ALT; (**L**) Hepatic Atg7 gene expression; (**M**) liver histology (H&E staining); (**N**) liver lipid accumulation (Oil Red O staining). Data are presented as mean ± SD, with significance indicated as described in text. Significant differences: * *p* < 0.05, ** *p* < 0.01, *** *p* < 0.001, **** *p* < 0.0001.

Acute ethanol exposure alone caused clear liver injury in mice. The liver index (liver weight/body weight ratio) was significantly increased (*p* < 0.01) while the spleen index was not significantly affected (Figure 1A,B). Ethanol-fed mice also exhibited hepatic lipid accumulation: hepatic total cholesterol and triglyceride levels were significantly elevated (*p* < 0.05), accompanied by an increase in malondialdehyde (MDA, a lipid peroxidation marker) (*p* < 0.05) (Figure 1C–E). In terms of inflammatory mediators, ethanol exposure led to significantly lower hepatic IL-10 and TNF-α levels (*p* < 0.01 vs. control) and a higher IL-1β level (*p* < 0.01) (Figure 1F–H). Additionally, serum markers of liver injury and gut permeability—LPS, AST, and ALT—were significantly elevated in ethanol-fed mice (*p* < 0.05) (Figure 1I–K). Consistently, histopathology revealed pronounced hepatic steatosis with abundant lipid droplets in the ethanol group (Figure 1M,N).

Introducing *E. faecalis* (E.f) produced a distinct pattern of effects. In terms of organ indices, *E. faecalis* supplementation significantly decreased the liver index in the absence of ethanol (CK + E.f vs. CK, *p* < 0.0001) but significantly increased it on the ethanol background (Et + E.f vs. Et, *p* < 0.001). In both backgrounds, E.f also caused a reduction in spleen index (relative to groups without E.f), indicating spleen atrophy (Figure 1A,B). Consistent with these changes, liver histology showed more severe damage in E.f-treated mice: even the CK + E.f group (no ethanol) developed noticeable hepatic steatosis with lipid droplet accumulation, and the Et + E.f group (ethanol plus E.f) had the most pronounced lesions (Figure 1M,N).

With regard to hepatic biochemistry, E.f did not further increase triglyceride or MDA levels, but it significantly lowered hepatic total cholesterol in both control and ethanol-treated mice (CK + E.f vs. CK and Et + E.f vs. Et, both *p* < 0.0001) (Figure 1C–E). *E. faecalis* exposure also led to higher hepatic Atg7 gene expression (Figure 1L), suggesting possible induction of autophagy. Despite the increase in autophagy (which can reduce hepatic cholesterol), the presence of E.f exacerbated fat accumulation in the liver, as evidenced by the worsened histology.

Inflammatory and injury markers displayed contrasting changes in the E.f-treated groups. In ethanol-fed mice, *E. faecalis* supplementation significantly reduced hepatic IL-1β levels compared to ethanol alone (Et + E.f vs. Et), bringing IL-1β below the level seen with ethanol by itself. Meanwhile, E.f had no additional effect on the already ethanol-suppressed IL-10 or TNF-α levels (Figure 1F–H). In the circulation, E.f markedly blunted the ethanol-induced increases in LPS and liver enzymes: in the Et + E.f group, serum LPS, AST, and ALT remained at levels comparable to those in the CK + E.f group, instead of rising as observed in the ethanol-only group (Figure 1I–K).

**Figure A1:**
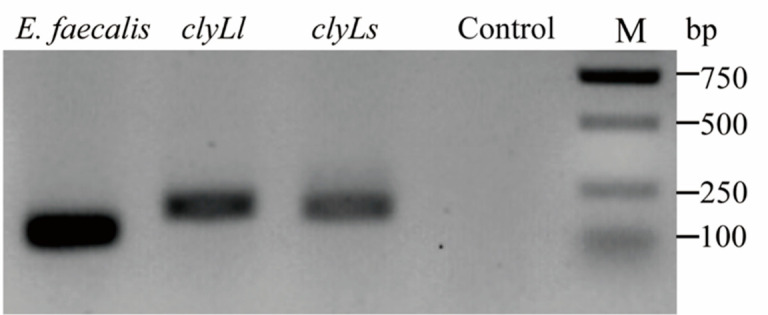
Agarose gel electrophoresis confirming the cytolysin gene in the *Enterococcus faecalis* strain used in this study. Lanes: *E. faecalis* (test strain), clyLI (cytolysin large subunit amplicon), clyLs (cytolysin small subunit amplicon), Control (no-template control). The sizes of the amplified fragments correspond to the expected lengths for cytolysin gene components. M, DNA size marker (bands indicated in base pairs). This confirms that the administered *E. faecalis* strain carries the functional cytolysin virulence determinant.

In summary, in this acute ethanol model, *E. faecalis* acted as a disease modifier that aggravated structural liver injury while paradoxically attenuating some inflammatory and injury markers. We confirmed that our *E. faecalis* strain carries the cytolysin virulence gene (Figure A1), and this strain is a well characterized cytolysin positive reference strain with documented hemolytic activity [21]. The observed increase in hepatic Atg7 expression may be associated with activation of autophagy, a process known to reduce hepatic cholesterol content but potentially promote neutral lipid storage—which could align with our findings of lower liver cholesterol yet worse steatosis in E.f-treated mice. Finally, although *E. faecalis* tended to reduce colonic tight-junction protein expression (especially Occludin) (Figure 4A–D), the short duration of exposure likely prevented significant gut leakage of endotoxin, which helps explain why the Et + E.f group did not develop elevated LPS or transaminases relative to ethanol alone (Figure 1I–K).

### 2.2. Enterococcus faecalis Aggravates Gut Microbiota Dysbiosis, with Campylobacterota Enrichment

**Figure 2:**
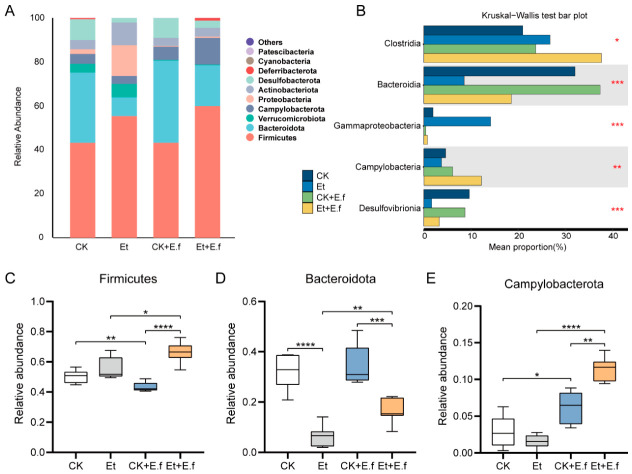
*Enterococcus faecalis* aggravates alcohol-induced gut microbiota dysbiosis. (**A**) Phylum-level composition of the cecal microbiota in control and treated groups. (**B**) Class-level taxa with significant abundance differences (top 5 classes) between groups. (**C**) Relative abundance of Firmicutes phylum; (**D**) Relative abundance of Bacteroidetes phylum; (**E**) Relative abundance of *Campylobacterota* phylum. Data are shown as mean percentages of total sequences ± SD. Asterisks indicate significant differences between the indicated groups (see text for *p* values). Significant differences: * *p* < 0.05, ** *p* < 0.01, *** *p* < 0.001, **** *p* < 0.0001.

16S rRNA gene sequencing of cecal contents revealed that the gut microbiota of control mice was dominated by members of the phyla *Firmicutes*, *Bacteroidota* (formerly *Bacteroidetes*), *Verrucomicrobiota*, and others (Figure 2A). Overall, ethanol administration led to a dysbiotic shift characterized by an increase in the relative abundance of *Firmicutes* and a decrease in *Bacteroidota* compared to controls (Figure 2A). At the class level, five taxa showed significant differences in abundance. Among these, *Clostridia*, *Bacteroidia*, *Gammaproteobacteria*, and *Desulfovibrionia* exhibited similar trends in both ethanol-treated groups (Et and Et + E.f) compared to their respective control groups (CK and CK + E.f). Specifically, the Et and Et + E.f groups had higher abundances of *Clostridia* and *Gammaproteobacteria* but lower abundances of *Bacteroidia* and *Desulfovibrionia* than the control groups (Figure 2B). Notably, the class *Campylobacteria* displayed divergent responses: its abundance decreased in the Et group (relative to CK), but increased significantly in the Et + E.f group (relative to CK + E.f) (Figure 2B), suggesting that *E. faecalis* supplementation promoted the growth of certain Campylobacteria under ethanol exposure.

Quantitative analysis of the dominant phyla reinforced these observations. The *Firmicutes*/*Bacteroidota* ratio was markedly elevated by ethanol. The relative abundance of *Firmicutes* was significantly higher in ethanol-treated mice (Et vs. CK, *p* < 0.0001; Et + E.f vs. CK + E.f, *p* < 0.0001), reaching the highest level in the Et + E.f group (Figure 2C). Conversely, *Bacteroidota* was significantly depleted by ethanol (Et vs. CK, *p* < 0.0001; Et + E.f vs. CK + E.f, *p* < 0.001) (Figure 2D). Thus, alcohol intake induced substantial gut microbiota dysbiosis, primarily characterized by an increased *Firmicutes*/*Bacteroidota* ratio. Moreover, *E. faecalis* supplementation further modified the microbiota: compared to ethanol alone, the Et + E.f group had an even higher *Firmicutes* abundance (*p* < 0.05), higher *Bacteroidota* abundance (*p* < 0.01), and a dramatically higher *Campylobacterota* abundance (*p* < 0.0001) (Figure 2C–E). In particular, the marked expansion of *Campylobacterota* in the Et + E.f group suggests that *E. faecalis* exacerbated the ethanol-induced microbial imbalance.

**Figure 3:**
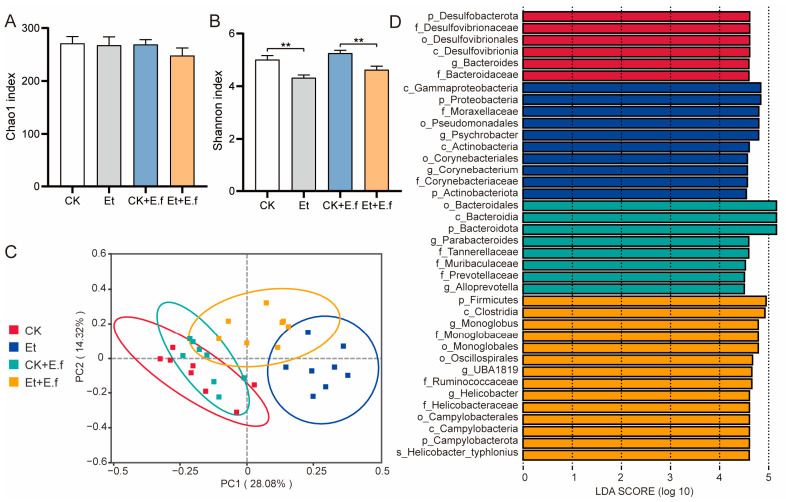
*E. faecalis* exacerbates gut microbial community perturbations. (**A**) Alpha-diversity measured by Chao1 richness index; (**B**) Alpha-diversity measured by Shannon index; (**C**) Principal Coordinates Analysis (PCoA) plot based on Bray–Curtis distances showing beta-diversity clustering of microbiota (PC1 28.08%, PC2 14.32% variation explained); (**D**) Linear discriminant analysis effect size (LEfSe) cladogram highlighting taxa enriched in each group (circles: phylum to genus level, with key discriminant taxa labeled). Significant differences: ** *p* < 0.01.

Next, we assessed microbial α- and β-diversity. The Chao1 richness index of the cecal microbiota was not significantly affected by ethanol or E.f treatment (Figure 3A). However, ethanol exposure led to a decrease in community diversity as measured by the Shannon index: both the Et group (vs. CK) and the Et + E.f group (vs. CK + E.f) had significantly lower Shannon diversity (*p* < 0.05) (Figure 3B). Notably, there were no significant differences in Shannon index between the presence or absence of E.f on the same background (Et vs. Et + E.f, and CK vs. CK + E.f), indicating that the reduction in diversity was primarily due to ethanol. Consistently, β-diversity analysis via principal coordinates analysis (PCoA) of Bray–Curtis distances showed that ethanol-fed mice (Et and Et + E.f) clustered separately from control mice, whereas the microbiota profiles of Et and Et + E.f mice were relatively closer to each other (Figure 3C). This suggests that ethanol exposure drove larger shifts in gut microbiota composition than did *E. faecalis* in this acute setting.

To identify specific bacterial taxa differentiating the groups, we performed linear discriminant analysis effect size (LEfSe) analysis. The LEfSe results revealed distinct taxa enriched in the presence or absence of *E. faecalis* under ethanol treatment (Figure 3D). In the Et group, the gut microbiota was enriched with *Psychrobacter* and *Corynebacterium*. In contrast, the Et + E.f group was enriched with *Monoglobus* and an uncultured genus *UBA1819* (both in phylum Firmicutes), as well as *Helicobacter typhlonius* (species, phylum Campylobacterota) (Figure 3D).

**Figure 4:**
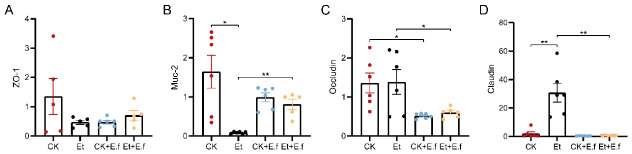
*E. faecalis* impairs colonic barrier function. (**A**–**D**) Relative mRNA expression levels of colonic tight junction proteins (including Occludin and other junctional molecules) in control vs. *E. faecalis*-colonized groups. Bars represent mean ± SD. Lower expression in *E. faecalis* groups (especially for Occludin) suggests a trend toward reduced gut barrier integrity. Significant differences: * *p* < 0.05, ** *p* < 0.01.

Furthermore, *E. faecalis* impacted the gut barrier integrity. Colon tissue from E.f-treated mice showed a trend toward lower expression of tight-junction proteins, with Occludin mRNA levels in particular reduced compared to controls (Figure 4A–D). This suggests that *E. faecalis* colonization may compromise the colonic epithelial barrier.

### 2.3. Akk11 Mitigates Intestinal Inflammation and Liver Damage

**Figure 5:**
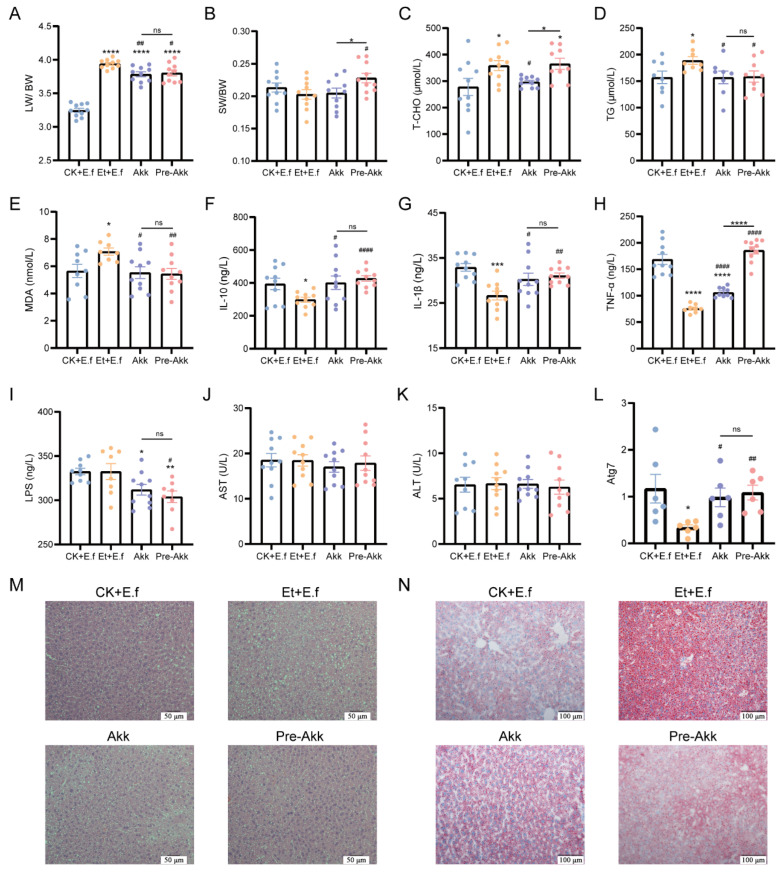
Akk11 supplementation alleviates *E. faecalis*- and ethanol-induced tissue damage. (**A**) Liver index; (**B**) Spleen index; hepatic (**C**) total cholesterol; (**D**) triglycerides; (**E**) malondialdehyde; (**F**) IL-10; (**G**) IL-1β; (**H**) TNF-α levels in liver; serum (**I**) LPS; (**J**) AST; (**K**) ALT; (**L**) Hepatic Atg7 expression; (**M**) liver histology (H&E staining); (**N**) liver Oil Red O staining. Data are mean ± SD. Significant differences between treatment groups and the Et + E.f model group are indicated (*p* < 0.05, *p* < 0.01, etc.), showing that Akk11 (especially post-treatment) mitigated hepatosplenomegaly, normalized liver lipids and cytokines, reduced LPS, and improved liver histology. * denotes significant differences between each group and the CK + E.f group, as well as between the Akk and Pre-Akk groups (* *p* < 0.05, ** *p* < 0.01, *** *p* < 0.001, **** *p* < 0.0001). # denotes significant differences between the Akk, Pre-Akk groups and the Et + E.f group (# *p* < 0.05, ## *p* < 0.01, #### *p* < 0.0001). ns, no significant difference.

We next evaluated whether supplementation with Akk11 could alleviate the damage caused by combined ethanol and *E. faecalis*. In terms of organ indices, Akk11 intervention significantly reduced ethanol/*E. faecalis*-induced liver enlargement. Both the Akk group (Akk given after disease induction, *p* < 0.01) and the Pre-Akk group (Akk given 2 days before ethanol exposure, *p* < 0.05) showed markedly lower liver index values compared to the untreated Et + E.f model group (Figure 5A). As for the spleen, *E. faecalis* had caused spleen atrophy (decreased spleen index) in this model; the Pre-Akk regimen significantly reversed this effect, restoring the spleen index toward normal levels (*p* < 0.01 vs. Et + E.f; Figure 5B). In contrast, therapeutic Akk11 treatment after disease onset did not significantly affect the spleen index (Figure 5B).

Akk11 supplementation also modulated hepatic lipid metabolism. Ethanol + *E. faecalis* exposure led to an increase in hepatic total cholesterol, which was significantly lowered by Akk treatment (*p* < 0.01; Figure 5C). The Pre-Akk group did not show a significant change in total cholesterol relative to Et + E.f (Figure 5C), possibly because the baseline *E. faecalis*-treated groups (CK + E.f and Et + E.f) already exhibited reduced cholesterol levels compared to groups without *E. faecalis*. Nonetheless, both Akk and Pre-Akk interventions improved other hepatic lipid indices. In both groups, hepatic triglyceride levels were significantly lower than in the untreated Et + E.f mice (*p* < 0.05), and hepatic MDA content was similarly reduced (Figure 5D,E).

Combined ethanol and *E. faecalis* exposure had suppressed several cytokines, and Akk11 affected these immune parameters as well. In the Et + E.f model, levels of inflammatory cytokines IL-1β and TNF-α, as well as the anti-inflammatory cytokine IL-10, were all reduced compared to controls; notably, IL-10 (*p* < 0.05) and TNF-α (*p* < 0.01) in the Et + E.f group were significantly lower than those in the CK + E.f group (Figure 5F–H). After Akk11 supplementation, these cytokine levels in both the Akk and Pre-Akk groups increased back to values comparable to the CK + E.f controls (Figure 5F–H), indicating a recovery of immune homeostasis. There were no significant differences between the Akk and Pre-Akk groups in IL-10 or IL-1β levels. However, the Pre-Akk group had a higher TNF-α level than the Akk group (*p* < 0.05) (Figure 5H), suggesting some differences in immune response depending on the timing of Akk11 administration.

**Figure 6:**
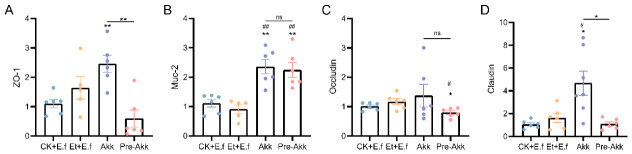
Akk11 strengthens the gut barrier after ethanol and *E. faecalis* exposure. (**A**–**D**) Relative expression of four colonic tight junction proteins in Et + E.f model mice versus those receiving Akk11 interventions. Akk11-treated mice (therapeutic Akk group) show increased expression of all measured tight junction markers, whereas the prophylactic Pre-Akk group shows a mild improvement. Enhanced tight junction expression is consistent with improved intestinal barrier integrity and reduced endotoxin leakage. * denotes significant differences between each group and the CK + E.f group, as well as between the Akk and Pre-Akk groups (* *p* < 0.05, ** *p* < 0.01). # denotes significant differences between the Akk, Pre-Akk groups and the Et + E.f group (# *p* < 0.05, ## *p* < 0.01). ns, no significant difference.

Akk11 intervention also impacted systemic endotoxin levels and liver enzymes. Both the Akk and Pre-Akk groups had lower serum LPS concentrations compared to the untreated Et + E.f group, with the Pre-Akk group exhibiting a more pronounced reduction in LPS (*p* < 0.05; Figure 5I). This finding suggests that Akk11 helped to preserve or restore gut barrier integrity, thereby reducing the translocation of endotoxin into the bloodstream. Consistent with this, colonic tight-junction protein expression was improved by Akk11 treatment. In Akk11-treated mice, the colonic mRNA levels of all four examined tight-junction proteins (including Occludin, ZO-1, etc.) were higher than in Et + E.f model mice, indicating an enhancement of barrier function. The Pre-Akk group also showed some increase in tight-junction protein expression, although the effect was more modest than in the Akk group (Figure 6A–D). On the other hand, Akk11 had no significant effect on serum AST or ALT levels (Figure 5J,K). This lack of change is likely because the presence of *E. faecalis* in the model had already reduced these liver enzymes to near-normal levels, thereby masking any potential further improvement by Akk11.

### 2.4. Akk11 Ameliorates Ethanol/E. faecalis-Induced Gut Dysbiosis and Barrier Dysfunction

**Figure 7:**
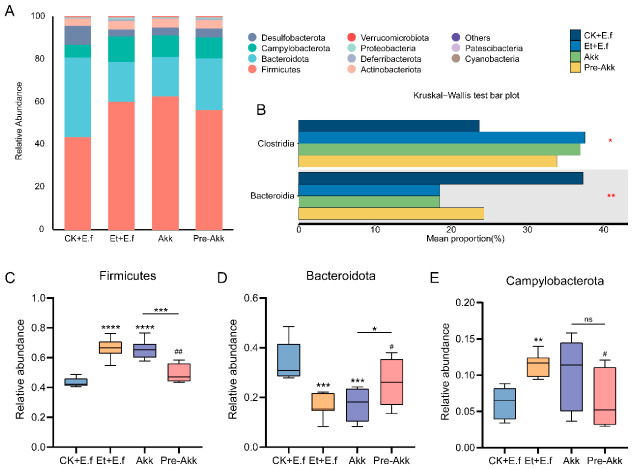
Akk11 intervention modulates the dysbiotic gut microbiota composition. (**A**) Phylum-level microbiota profiles in ethanol/*E. faecalis* model mice and those with Akk11 treatment. (**B**) Class-level comparison of two major taxa (*Clostridia* and *Bacteroidia*) showing significant differences; ethanol increases *Clostridia* and decreases *Bacteroidia*, which Pre-Akk partially reverses. (**C**) Relative abundance of *Firmicutes* phylum; (**D**) *Bacteroidetes* phylum; (**E**) *Campylobacterota* phylum under different treatments. Prophylactic Akk11 (Pre-Akk) significantly shifts these abundances toward normal (lower *Firmicutes* and *Campylobacterota*, higher *Bacteroidetes*), whereas therapeutic Akk11 after modeling shows no significant change. Statistical significance is indicated for Pre-Akk vs. untreated model comparisons (*p* < 0.05, *p* < 0.01). * denotes significant differences between each group and the CK + E.f group, as well as between the Akk and Pre-Akk groups (* *p* < 0.05, ** *p* < 0.01, *** *p* < 0.001, **** *p* < 0.0001). # denotes significant differences between the Akk, Pre-Akk groups and the Et + E.f group (# *p* < 0.05, ## *p* < 0.01). ns, no significant difference.

In terms of gut microbiota composition, Akk11 administration yielded different outcomes depending on the timing of intervention. The combined ethanol + E.f treatment had created a dysbiotic state characterized by low *Bacteroidota* and high *Firmicutes* abundance. This imbalance persisted in the Akk treatment group but was partially corrected in the Pre-Akk group. Specifically, prophylactic Akk11 significantly reduced the *Firmicutes* abundance (*p* < 0.01) while increasing *Bacteroidota* abundance (*p* < 0.05) relative to the untreated Et + E.f condition (Figure 7A). The Pre-Akk group also exhibited a significantly lower relative abundance of *Campylobacterota* (*p* < 0.05) compared to the Et + E.f group, a change not observed in the post-treatment Akk group (Figure 7C–E). By contrast, administering Akk11 after disease onset (Akk group) did not significantly alter the major phylum-level proportions (*Firmicutes* or *Bacteroidota*) or the level of *Campylobacterota* compared to the untreated Et + E.f group.

At the class level, ethanol exposure led to an increase in the relative abundance of Clostridia and a decrease in *Bacteroidia* in the gut microbiota. The Akk treatment did not significantly change this dysbiotic pattern, as *Clostridia* remained elevated and *Bacteroidia* remained low (similar to Et + E.f) (Figure 7B). In contrast, the Pre-Akk intervention markedly counteracted these ethanol-induced shifts: the Pre-Akk group had a significantly lower *Clostridia* abundance (*p* < 0.05) and a higher *Bacteroidia* abundance (*p* < 0.01) compared to the Et + E.f group (Figure 7B). These results indicate that initiating Akk11 prior to ethanol exposure was more effective in modulating the gut microbial community than administering it after dysbiosis had already developed.

**Figure 8:**
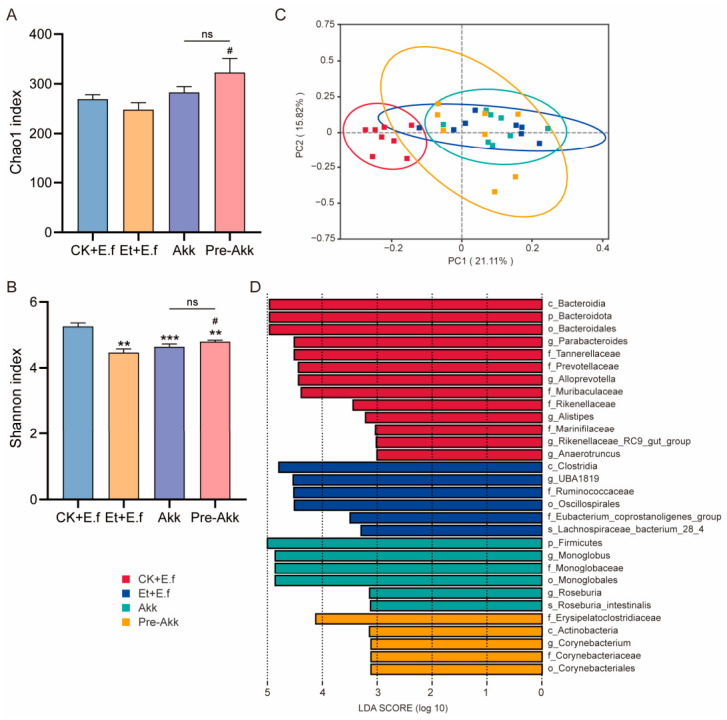
Akk11 improves gut microbial diversity and community structure. (**A**) Chao1 richness index of cecal microbiota in each group; (**B**) Shannon diversity index; (**C**) PCoA plot of Bray–Curtis distances illustrating differences in community composition between groups (each point represents one mouse’s microbiota; percentage variation explained by each axis is indicated); (**D**) LEfSe analysis identifying taxa differentially enriched in the Akk and Pre-Akk groups compared to untreated dysbiotic mice. Prophylactic Akk11 increases both richness and diversity (**A**,**B**) and yields a microbiota composition distinct from the untreated ethanol group (**C**), with specific beneficial or commensal taxa enriched (**D**). * denotes significant differences between each group and the CK + E.f group, as well as between the Akk and Pre-Akk groups (** *p* < 0.01, *** *p* < 0.001). # denotes significant differences between the Akk, Pre-Akk groups and the Et + E.f group (# *p* < 0.05). ns, no significant difference.

Microbial diversity analyses highlighted the impact of Akk11 timing on the gut ecosystem. The Shannon diversity index of the gut microbiota was significantly reduced in the Et + E.f group (*p* < 0.01), Akk group (*p* < 0.001), and Pre-Akk group (*p* < 0.01) compared to the CK + E.f control, indicating that ethanol (with or without *E. faecalis* or Akk11) lowered overall microbial diversity (Figure 8B). However, importantly, the Pre-Akk group showed a significant increase in both the Chao1 richness index and the Shannon diversity index compared to the untreated Et + E.f group (*p* < 0.05 for both; Figure 8A,B). This suggests that early intervention with Akk11 preserved a greater number of total and low-abundance bacterial species, thereby maintaining higher gut microbiota richness and evenness despite ethanol exposure. Notably, this protective effect on diversity is consistent with observations from our previous obesity model [28], underscoring Akk11’s capacity to beneficially modulate the gut microbiome (Figure 8A,B).

β-diversity analysis by principal coordinates analysis (PCoA, Bray–Curtis distance) further illustrated the differences between groups. The gut microbial communities of the CK + E.f vs. Et + E.f mice clustered distinctly from each other, with the first two principal coordinates explaining 21.11% and 15.82% of the variance, respectively (Figure 8C). This clear separation indicates substantial alterations in community structure due to ethanol exposure. Among the Akk-treated groups, the Pre-Akk group clustered apart from the Akk group, suggesting that prophylactic Akk11 had a more pronounced effect on reshaping the gut microbiota composition than treatment with Akk11 after disease induction (Figure 8C).

LEfSe analysis was applied to pinpoint specific taxa that were differentially enriched following Akk11 intervention. The Akk group microbiota was enriched in *Monoglobus* (genus, phylum *Firmicutes*) and in *Roseburia intestinalis* (species, phylum *Firmicutes*). In contrast, the Pre-Akk group showed enrichment of an *Erysipelatoclostridiaceae* family taxon (phylum *Firmicutes*) and genus *Corynebacterium* (phylum *Actinobacteriota*), among other differences (Figure 8D). These distinctions further demonstrate that the timing of Akk11 supplementation leads to different microbial signatures in the gut.

### 2.5. Mechanisms of Helicobacter typhlonius and Bacteroides sartorii in the Gut–Liver Axis and Protective Effect of Akk11

**Figure 9:**
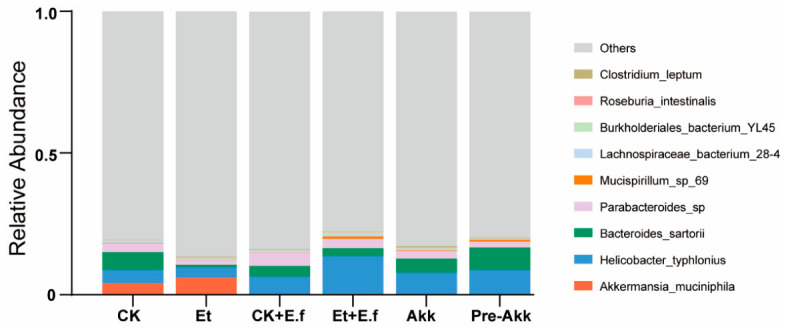
Akk11 modulates specific gut microbes involved in the gut–liver axis.

To further elucidate the microbial mechanisms underlying the gut–liver axis in this model, we analyzed species-level changes in key taxa linked to intestinal inflammation and liver injury (Figure 9). Ethanol feeding combined with *E. faecalis* colonization (Et + E.f group) markedly enriched *Helicobacter typhlonius* (a *Campylobacterota* linked to intestinal inflammation via innate lymphoid cell regulation) in the gut. This overgrowth likely bridges *E. faecalis*-aggravated dysbiosis and gut-liver inflammation, as *Helicobacter* species activate mucosal immunity. Notably, Akk11 supplementation (especially the Pre-Akk group) drastically reduced *H. typhlonius* abundance—nearly eliminating it in Pre-Akk mice—suggesting early Akk11 intervention prevents this inflammatory microbe’s bloom, thereby dampening associated intestinal inflammation and downstream liver injury.

Alcohol exposure also depleted beneficial *Bacteroides sartorii* (a commensal with probiotic potential) in Et/Et + E.f groups, while Akk11 (most effectively Pre-Akk) restored its abundance to near-control levels. This recovery likely supports gut-liver health: *B. sartorii* may produce anti-inflammatory metabolites or preserve barrier integrity, and its specific enrichment implies Akk11 targets key beneficial taxa rather than broadly stimulating *Bacteroides* species.

Additionally, ethanol feeding elevated the opportunistic pathogen *Burkholderiales bacterium YL45* (linked to inflammatory conditions), while *E. faecalis* had no further effect on its abundance. Only prophylactic Akk11 (Pre-Akk group) suppressed *YL45* to near-baseline levels (therapeutic Akk showed no significant reduction), highlighting the importance of early intervention in controlling this pro-inflammatory bacterium.

In summary, *E. faecalis* exacerbates alcohol-induced liver injury partly by worsening dysbiosis (promoting *H. typhlonius* and other pathobionts) and impairing the gut barrier. Akk11 counteracts these effects via coordinated mechanisms: reinforcing barrier integrity, reshaping microbiota (boosting *B. sartorii* and curbing *H. typhlonius*/*YL45*), and mitigating inflammation—ultimately reducing endotoxemia, normalizing cytokines, and improving liver histopathology in treated mice.

## 3. Discussion

Building upon prior clinical and experimental evidence that links *Enterococcus faecalis*, particularly cytolysin-positive strains, to the severity of alcoholic hepatitis [21], our study confirms and extends this finding in a controlled mouse model of acute ethanol exposure. We not only corroborate the pathogenic role of *E. faecalis* but also delineate a complex, paradoxical pattern of host response and uncover novel mechanistic insights into how it exacerbates liver injury through gut-level actions.

A pivotal finding of our work is the dual role of *E. faecalis* in acute ALD. While it unequivocally worsened structural liver damage, as evidenced by a higher liver index and more severe histopathological steatosis, it concurrently attenuated several circulating and hepatic inflammatory markers. The reduction in serum LPS, AST, and ALT in the Et + E.f group, despite worse histological injury, is an intriguing but unresolved observation. One hypothesis is that *E. faecalis* may trigger compensatory or adaptive responses that mask the classical signs of hepatocyte damage in the acute phase. The significant upregulation of hepatic Atg7 may indicate induction of autophagy [29,30], a process known to reduce hepatic cholesterol content (consistent with our data) but potentially promote neutral lipid storage, aligning with the observed dissociation between biochemical and histological measures of steatosis. We acknowledge that autophagy activation was inferred solely from Atg7 mRNA expression, and future studies measuring LC3-II or p62 are needed to confirm functional autophagic flux [31,32]. This novel finding underscores the complexity of host–pathogen interactions and highlights the importance of using multiple complementary endpoints to assess liver injury, as reliance on standard serum biomarkers alone could be misleading. Direct measurements of gut permeability (e.g., FITC-dextran) are also needed to validate the hypothesized link between barrier changes and endotoxemia [33,34].

Our data further elucidate how the gut microbiota and the gut-liver axis may mediate *E. faecalis* pathogenicity. Beyond confirming that *E. faecalis* aggravates ethanol-induced dysbiosis, we identified a specific and dramatic expansion of the phylum *Campylobacterota* as a key microbial signature. Species-level analysis pinpointed *Helicobacter typhlonius*, a known activator of mucosal immunity, as a taxon enriched in the Et + E.f group. This enrichment is associated with worsened liver injury, suggesting a possible link; however, causality cannot be inferred from correlational data. Concurrently, *E. faecalis* impaired the colonic epithelial barrier, as shown by the downregulation of tight-junction proteins like Occludin at the mRNA level. We note that these conclusions are based primarily on mRNA expression, and protein level validation (e.g., Western blot or quantitative IHC) would be necessary to confirm functional barrier changes. Although this did not result in elevated serum LPS in our acute model, it may prime the host for increased bacterial translocation upon prolonged injury.

The therapeutic potential of Akk11 was clearly demonstrated by its ability to mitigate the liver injury and metabolic disturbances induced by the combination of ethanol and *E. faecalis*. Akk11 administration reduced hepatomegaly, lowered hepatic triglycerides and oxidative stress, and normalized the levels of key inflammatory cytokines. A pivotal mechanism behind this protection was the reinforcement of the intestinal barrier, as indicated by the increased expression of colonic tight-junction proteins and the consequent reduction in systemic endotoxemia. This direct barrier-strengthening effect is a well-established property of *A. muciniphila* and appears to be a cornerstone of its efficacy in this model.

Notably, the timing of Akk11 intervention was a critical determinant of its impact on the gut microbial community. Prophylactic administration (Pre-Akk) was superior to therapeutic treatment in reshaping the dysbiotic microbiota. Pre-Akk intervention significantly normalized the ethanol-elevated *Firmicutes*/*Bacteroidota* ratio, reduced the abundance of the deleterious *Campylobacterota* and *H. typhlonius*, and enhanced overall microbial richness and diversity. In contrast, post-onset Akk11 treatment had a more modest effect on community structure. This suggests that administering Akk11 *before* the onset of dysbiosis helps to stabilize the gut ecosystem, making it more resilient to the disruptive effects of ethanol and *E. faecalis* [35].

Further elucidating the microbial dynamics, we found that Akk11’s protection was associated with the targeted modulation of specific bacteria. It was associated with reduced abundance of *H. typhlonius* and *Burkholderiales* bacterium YL45, and with restored abundance of *Bacteroides sartorii*. These associations do not imply causation, and functional studies (e.g., gavage with pure cultures or fecal transfer) are needed to establish causal roles for these specific bacteria [36,37,38]. Nonetheless, the consistent patterns observed across independent groups suggest that Akk11 facilitates a shift in the ecological balance towards a more beneficial state.

In addition to probiotic strategies, bacteriophage therapy has emerged as a promising approach to target enteric pathogens such as *E. faecalis* in ALD [39,40]. Our findings complement this literature by showing that a next generation probiotic can also counteract *E. faecalis* exacerbated injury through microbiota remodeling and barrier protection.

Although the present study did not directly investigate the gut–brain axis, the observed reinforcement of intestinal barrier function by *A. muciniphila* may have implications for brain health. A compromised intestinal barrier can lead to systemic low-grade inflammation and microbial metabolite translocation, both of which are increasingly recognized as contributors to neuroinflammation and behavioral disorders via the gut–brain axis [41,42,43]. Beneficial bacteria represented by *A. muciniphila* can restore tight junction structure and alleviate endotoxemia. Such strains exert protective effects on the liver and intestine, and are promising for the prevention or alleviation of various neuropsychiatric complications induced by hepatic and intestinal disorders, including anxiety, depression and cognitive impairment [44,45,46].

## 4. Materials and Methods

### 4.1. Bacterial Strains and Culture

*Enterococcus faecalis* ATCC29212 (cytolysin-positive clinical isolate, stored in this laboratory) was cultured aerobically in Brain Heart Infusion (BHI) broth at 37 °C for 18–24 h. *Akkermansia muciniphila* (strain Akk11, supplied by Wecare Probiotics Co., Ltd., Suzhou, China) was isolated from infant feces and cultured anaerobically at 37 °C for approximately 48 h in a mucin-based basal medium, as described previously [47]. Bacterial cells from both cultures were harvested by centrifugation (8000× *g*, 4 °C, 10 min), washed twice with sterile phosphate-buffered saline (PBS), and resuspended in sterile PBS for administration. The presence of the cytolysin gene (cyl) in our *E. faecalis* strain was confirmed by PCR (Figure A1).

### 4.2. Animal Experimental Design

Male C57BL/6 mice (6–7 weeks old) were purchased from the Experimental Animal Center of Huazhong Agricultural University and housed under specific pathogen-free conditions (12-h light/dark cycle, 22–24 °C, 50–60% humidity) with free access to standard chow and water. After one week of acclimatization, mice were randomly assigned to six experimental groups (*n* = 8–10 per group) using a computer-generated random number sequence (Excel, RAND function). Group allocation was concealed in opaque envelopes until the start of the experiment. The groups were: (1) Control (CK), (2) *E. faecalis* alone (CK + E.f), (3) Ethanol alone (Et), (4) Ethanol + *E. faecalis* (Et + E.f), (5) Ethanol + *E. faecalis* + therapeutic Akk11 (Akk), (6) Ethanol + *E. faecalis* + prophylactic Akk11 (Pre-Akk). The prophylactic Akk11 group received Akk11 for 2 days prior to ethanol exposure, while the therapeutic group received it after ethanol/*E. faecalis* challenge. All bacterial suspensions were administered by oral gavage at a dose of 1 × 10^9^ colony-forming units (CFU) in 200 µL PBS daily. Control groups received an equal volume of sterile PBS. Sample size was not determined by a formal power analysis; the number of animals per group (*n* = 8–10) was chosen based on our previous studies and the published literature to detect a biologically meaningful difference in serum ALT and hepatic TG levels (estimated effect size > 30%), while minimizing animal use. No animals were excluded from the analysis. All experimental animals have received approval from the Animal Ethics Committee of the Animal Experimental Ethical Inspection of Laboratory Animal **Centre**, Huazhong Agriculture University (Ethics Approval Number: HZAUMO-2024-0272, approved on 7 July 2022).

### 4.3. Acute Ethanol Exposure Model

Acute alcohol-associated liver injury was induced by a single oral gavage of ethanol (5 g/kg body weight, 31.5% *v*/*v* in PBS) after overnight fasting. Control mice received an isocaloric maltose solution. Mice were sacrificed 9 h after ethanol administration.

### 4.4. Sample Collection and Organ Index Calculation

Blood was collected from the retro-orbital plexus, and serum was separated by centrifugation (3000× *g*, 15 min, 4 °C). Liver and spleen were excised and weighed immediately. The liver index and spleen index were calculated as (organ weight/body weight) × 100%.

### 4.5. Biochemical and Cytokine Analysis

Hepatic total cholesterol (TC), triglycerides (TG), and malondialdehyde (MDA) were measured using commercial assay kits (Nanjing Jiancheng Bioengineering Institute, Nanjing, China) according to the manufacturer’s protocols. Serum alanine aminotransferase (ALT), aspartate aminotransferase (AST), and lipopolysaccharide (LPS) levels were quantified using corresponding ELISA kits (Cusabio, Wuhan, China). Hepatic levels of interleukin-10 (IL-10), interleukin-1β (IL-1β), and tumor necrosis factor-α (TNF-α) were measured by multiplex immunoassay (Bio-Plex, Bio-Rad, Hercules, CA, USA) according to the manufacturer’s instructions.

### 4.6. Histopathological Evaluation

Liver tissues were fixed in 4% paraformaldehyde, embedded in paraffin, and sectioned. Sections were stained with Hematoxylin and Eosin (H&E) for general morphology and with Oil Red O (ORO) for neutral lipid visualization. Histological scoring of steatosis was performed by a pathologist blinded to the grouping. Colon tissues were processed similarly for H&E staining and immunohistochemical analysis of tight junction proteins (Occludin, ZO-1). Images were captured using a light microscope (Nikon Eclipse 80i, Tokyo, Japan).

### 4.7. Gut Permeability and Tight Junction Gene Expression

Colon tissue mRNA was extracted using RNA Easy™ Animal RNA Isolation kit (Beyotime, Shanghai, China), and cDNA was synthesized using a reverse transcription kit (Takara, Tokyo, Japan). Quantitative real-time PCR (qRT-PCR) was performed on a Quantstudio (Thermo Fisher Scientific, Waltham, MA, USA) with SYBR Green Master Mix (Vazyme, Nanjing, China). Primer sequences for Occludin, ZO-1, Claudin, and MUC-2 were designed and normalized to β-actin. Relative gene expression was calculated using the 2^−ΔΔCt^ method.

### 4.8. 16S rRNA Gene Sequencing and Bioinformatics Analysis

Upon collection, cecal samples were immediately snap-frozen in sterile cryovials and maintained at −80 °C for subsequent DNA extraction. Total genomic DNA was isolated from the cecal contents using a commercial kit (Tiangen Biotech, Beijing, China). The concentration and purity of the extracted DNA were assessed with a NanoDrop 2000 spectrophotometer (Thermo Fisher Scientific, Massachusetts, USA). The V4 region of the bacterial 16S rRNA gene was then amplified via polymerase chain reaction (PCR) using the primers 515F (5′-GTGCCAGCMGCCGCGGTAA-3′) and 806R (5′-GGACTACHVGGGTWTCTAAT-3′). The resulting PCR amplicons were pooled, purified, and subjected to end-repair, A-tailing, and adapter ligation to construct sequencing libraries. Library quantification and quality control were performed using Qubit fluorometry and quantitative PCR. Qualified libraries were sequenced on an Illumina NovaSeq 6000 (Illumina, San Diego, CA, USA) system using a PE250 strategy. After sequencing, paired-end reads were merged with FLASH (v1.2.11) and quality-filtered using fastp (v0.23.1). High-quality sequences were clustered into amplicon sequence variants (ASVs) at 99% similarity threshold using DADA2. Taxonomic annotation of ASVs was carried out against the SILVA 138.1 database. Alpha diversity indices were calculated with QIIME2, while beta diversity was evaluated based on Bray–Curtis distances and visualized through principal coordinate analysis (PCoA). Differences in microbial community structure among groups were statistically tested using permutational multivariate analysis of variance (PERMANOVA) with 999 permutations and analysis of similarities (ANOSIM). For alpha diversity comparisons, Kruskal–Wallis test followed by Dunn’s post hoc test with Benjamini–Hochberg correction for multiple comparisons was applied. Taxon-specific biomarkers were identified via linear discriminant analysis effect size (LEfSe) with an LDA score threshold > 2.0 and *p* < 0.05. Functional profiling of the microbiota was inferred using PICRUSt2.

### 4.9. Statistical Analysis

All data are expressed as mean ± standard deviation (SD). Statistical comparisons among multiple groups were performed by one-way analysis of variance (ANOVA) followed by Tukey’s post hoc test using GraphPad Prism 8.00. For microbiota beta diversity, permutational multivariate analysis of variance (PERMANOVA) was applied. A *p*-value < 0.05 was considered statistically significant (* *p* < 0.05, ** *p* < 0.01, *** *p* < 0.001, **** *p* < 0.0001).

## 5. Conclusions

In conclusion, our findings delineate a pathogenic pathway in acute ALD wherein *E. faecalis* exacerbates liver injury by worsening microbiota dysbiosis—particularly through the enrichment of *H. typhlonius*—and impairing intestinal barrier function. The next-generation probiotic *A. muciniphila* (Akk11) effectively counteracts these effects through a coordinated mechanism that includes barrier reinforcement, microbiota remodeling, and inflammation mitigation. The superior efficacy of prophylactic Akk11 administration highlights the importance of early microbial intervention in preventing the breakdown of gut-liver axis homeostasis. These results position Akk11 as a promising, microbiota-targeted candidate for the prevention and treatment of ALD.

## Data Availability

The datasets presented in this study can be found in online repositories. The raw sequence data are available in the NCBI Sequence Read Archive (SRA) under accession number PRJNA1378506. All other data supporting the findings of this study are provided within the article and its Appendix A and Appendix B.

## Appendix B

**Primers used in this study**

**Primers**	**Sequence (5′ to 3′)**
clyLl-F	ATGGAAAATTTAAGTGTAGTTCCTAG
ClyLl-R	TTAACAATGTTTTAAAGACACAACTAC
ClyLs-F	GTGCTAAATAAGGAAAATCAAGAAAAC
ClyLs-R	TTAGCAAAATTTAGCTGAAAATAATGC
Ef-F	CGCTTCTTTCCTCCCGAGT
Ef--R	GCCATGCGGCATAAACTG
Atg7-F	TGGCTGCTACTTCTGCAATGAT
Atg7-R	CAGGACAGAGACCATCAGCTCC
*Occludin*-F	TGGCTATGGAGGCGGCTATGG
*Occludin*-R	AAGGAAGCGATGAAGCAGAAGGC
*Claudin*-F	GCTGGGTTTCATCCTGGCTTCTC
*Claudin*-R	CCTGAGCGGTCACGATGTTGTC
*ZO-1*-F	GCGAACAGAAGGAGCGAGAAGAG
*ZO-1*-R	GCTTTGCGGGCTGACTGGAG
*MUC2*-F	GAAGCCAGATCCCGAAACCA
*MUC2*-R	TGTAGGAGTCTCGGCAGTCA
*β-actin*-F	CTACCTCATGAAGATCCTGACC
*β-actin*-R	CACAGCTTCTCTTTGATGTCAC
